# A unified approach for including non-extractable residues (NER) of chemicals and pesticides in the assessment of persistence

**DOI:** 10.1186/s12302-018-0181-x

**Published:** 2018-12-17

**Authors:** Andreas Schäffer, Matthias Kästner, Stefan Trapp

**Affiliations:** 1 Institute for Environmental Research, RWTH Aachen University, Worringerweg 1, 52074 Aachen, Germany; 2 Department Environmental Biotechnology, Helmholtz Centre for Environmental Research, UFZ, Permoserstraße15, 04318 Leipzig, Germany; 3 Department of Environmental Engineering, Technical University of Denmark, Bygningstorvet bd. 115, 2800 Kongens Lyngby, Denmark

## Abstract

All chemicals form non-extractable residues (NER) to various extents in environmental media like soil, sediment, plants and animals. NER can be quantified in environmental fate studies using isotope-labeled (such as ^14^C or ^13^C) tracer compounds. Previous NER definitions have led to a mismatch of legislation and state of knowledge in research: the residues are assumed to be either irreversibly bound degradation products or at least parts of these residues can be released. In the latter assumption, soils and sediments are a long-term source of slowly released residues. We here present a conceptual experimental and modeling approach to characterize non-extractable residues and provide guidance how they should be considered in the persistence assessment of chemicals and pesticides. Three types of NER can be experimentally discriminated: sequestered and entrapped residues (type I), containing either the parent substance or xenobiotic transformation products or both and having the potential to be released, which has indeed been observed. Type II NER are residues that are covalently bound to organic matter in soils or sediments or to biological tissue in organisms and that are considered being strongly bound with very low remobilization rates like that of humic matter degradation rates. Type III NER comprises biogenic NER (bioNER) after degradation of the xenobiotic chemical and anabolic formation of natural biomolecules like amino acids and phospholipids, and other biomass compounds. We developed the microbial turnover to biomass (MTB) model to predict the formation of bioNER based on the structural properties of chemicals. Further, we proposed an extraction sequence to obtain a matrix containing only NER. Finally, we summarized experimental methods to distinguish the three NER types. Type I NER and type II NER should be considered as potentially remobilizable residues in persistence assessment but the probability of type II release is much lower than that of type I NER, i.e., type II NER in soil are “operationally spoken” irreversibly bound and can be released only in minute amounts and at very slow rates, if at all. The potential of remobilization can be evaluated by chemical, physical and biological methods. BioNER are of no environmental concern and, therefore, can be assessed as such in persistence assessment. The general concept presented is to consider the total amount of NER minus potential bioNER as the amount of xenoNER, type I + II. If a clear differentiation of type I and type II is possible, for the calculation of half-life type I NER are considered as not degraded parent substance or transformation product(s). On the contrary, type II NER may generally be considered as (at least temporarily) removed. Providing proof for type II NER is the most critical issue in NER assessment and requires additional research. If no characterization and additional information on NER are available, it is recommended to assess the total amount as potentially remobilizable. We propose our unified approach of NER characterization and evaluation to be implemented into the persistence and environmental hazard assessment strategies for REACH chemicals and biocides, human and veterinary pharmaceuticals, and pesticides, irrespective of the different regulatory frameworks.

**Electronic supplementary material:**

The online version of this article (10.1186/s12302-018-0181-x) contains supplementary material, which is available to authorized users.

## Introduction

Criteria for the assessment of chemical properties and toxicological and environmental behavior of industrial chemicals in general, and particularly for biocidal products, plant protection products, and veterinary medicines are summarized in specific European legislations, [1–3] and guidelines [4–6], respectively. For the assessment of PBT properties under REACH, there are guidance documents available [7–9], which provide principles on the assessment of NERs. However, currently, there is no detailed description of the extraction techniques to differentiate NER types available. As there is no unified guidance available for the differentiation of different NER types in the general regulatory context, here, an approach is suggested based on a thorough review of the current scientific state of the art. Our paper is based on an extensive discussion paper on the interpretation of non-extractable residues in degradation assessment, which we would like to present to a wider audience [10].

Besides various degradation and transport processes, all chemicals that enter the environment form NER in solid matrices in varying amounts [11, 12]. NER formation can be quantified in environmental fate studies using, e.g., ^14^C or ^13^C-labeled tracer compounds. Previous NER definitions led to a mismatch of legislation and current state of knowledge in research and modeling; only parent compounds and primary metabolites are considered as NER, whereas remaining label conversion into natural bio-components was explicitly excluded [13]. The regulatory views on NER formation differ considerably with the two extremes of (i) assuming them as either degraded residues of no environmental concern in the regulation of pesticides [14, 15], at least if the NER are below or the mineralisation rates above certain threshold values, or (ii) as potentially bioavailable and non-degraded residues (“parent substance”) in the regulation of general industrial chemicals [7, 9, 16] if no clear indication for ultimate degradation or irreversible immobilization is available. In other words, NER in the respective matrix are valued either as ‘safe sink’ or as potential ‘hidden hazard’.

We will argue why the extreme views of NER as degraded versus non-degraded and bioavailable residues have to be reconsidered and why it is necessary to distinguish degradation of chemicals from dissipation, which is only possible by characterizing the underlying mechanisms. A conceptual framework and an analytical toolbox for the characterization of NER formation as well as potential approaches for the assessment of the NER stability together with further research needs are provided.

## What is known

Chemicals entering the environment undergo various abiotic and biotic turnover processes [17, 18], are taken up by living organisms, leach to the groundwater, and volatilize to the atmosphere, but a part of the chemical will always be immobilized as NER [19], i.e., fractions immobilized to solid matrices. Although these processes have been investigated for decades, the formation of NER in soils, sediments and biological tissue [20–22] is often considered ‘black box’ in environmental risk assessment of chemicals. Usually, NER were in the past only characterized with respect to the percentage of radioactivity associated with fulvic acid, humic acid and humin fractions of organic matter [11, 19, 20, 22–26] but underlying mechanisms of binding were only partially understood except that covalent binding to humic matter were qualitatively differentiated from non-covalent interactions like electrostatic interactions, hydrogen bonding, van der Waals forces, and hydrophobic interactions [25, 27–30].

### Definition of NER

According to the mostly cited IUPAC definition [13], NER in soils are defined as species originating from chemicals that remain un-extracted by methods which do not significantly change the chemical nature of these residues. Non-extractable residues are considered to exclude fragments recycled through metabolic pathways leading to natural products. Later, it was stated [31]: “Bound residues represent compounds in soils, plants, or animals which persist in the matrix in the form of the parent substance or its transformation product(s) after extraction. The extraction method must not substantially change the compounds themselves or the structure of the matrix”. However, both definitions cause potential for misunderstanding and misinterpretation: they focus on not altering the matrix, which cannot be excluded by many applied methods (see below), and the definition of Führ [31] is not considering the formation of biogenic NER. Harsher chemical or physical environmental processes such as soil acidification may alter the matrix and may also change the nature of the xenobiotic and its binding mechanism.

Since NER have to be quantified by radio isotope labeling (^14^C) of a chemical at the most stable part of the molecule [32], the detection can only be related to the labelled atom and not to molecular speciation. Thus, the structural identity of NER remains unknown. Studies under identical experimental conditions, in which the xenobiotic is isotope labeled at different positions, lead to different results regarding mineralization, degradation half-lives and NER formation depending on the stability of the labelled molecular moiety. Only by specific spectroscopic techniques after labeling the molecule with suitable stable isotopes, e.g., ^13^C or ^15^N for corresponding NMR analysis [33–36] or high-resolution MS [37–43] or ^14^C-labeling combined with LC–MS [44], structural features of NER have been elucidated, however, often at elevated concentrations of the test substances.

**Fig. 1:**
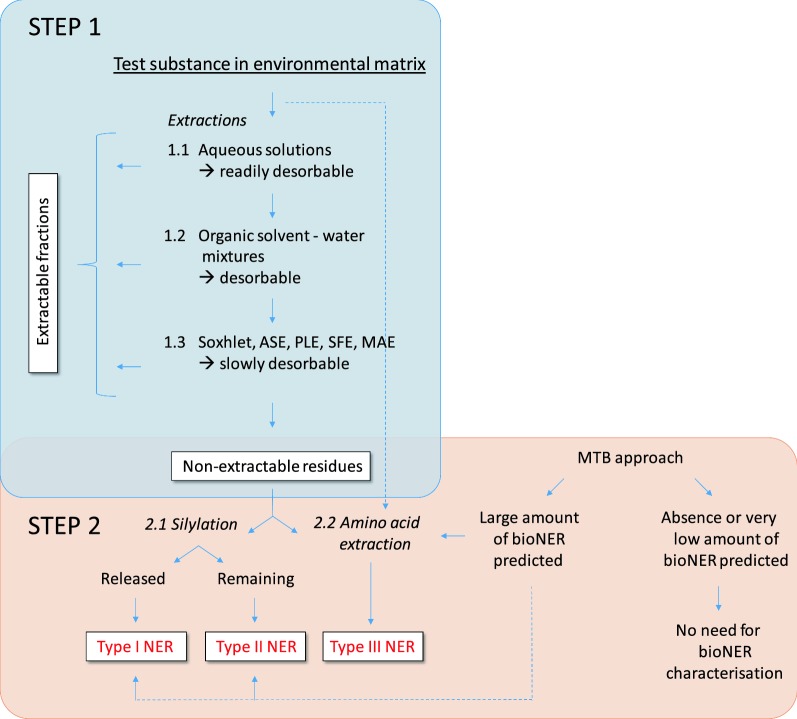
Extraction scheme. Proposed scheme of extraction steps for deriving extractable fractions and investigating NER. Alkaline humic matter extraction can be used as an alternative to direct silylation of the NER containing matrix, e.g., soil. Then, each humic matter fraction (fulvic and humic acids and humin) derived by alkaline extraction has to be silylated to enable the differentiation of type I and type II NER. Amino acid extraction can be additionally performed with the whole sample prior to any extraction. The difference to the amount of bioNER can be considered as the amount of labelled biomolecules that may be extracted in the step 1 procedures

Recently, the state of the art regarding NER was reviewed and various types of NER were classified [12]. It was concluded that the total amount of NER is the sum of strongly adsorbed or entrapped (type I)—both may be considered as sequestered—and covalently bound residues (type II) both either derived from the parent substance or from transformation or degradation products; a third type (III) refers to biogenic NER that are derived from biotic degradation (see Fig. 1 and Table 1). This degradation results in label transformation to various biomolecules, e.g., amino acids, phospholipids, etc., which has been shown by stable isotope labeling (^13^C, ^15^N) [37, 38, 40–43]. The three NER types are formed by competing processes [12]. Below, we present discriminating analytical methods in the proposal for an extraction scheme.

**Table 1:** Types of NER, related properties, and methods for identification

NER types	I [24, 45–48]	II [34, 47, 49–52]	III [38, 40, 41, 53–55]
Properties	Strongly sorbed, entrapped (both = sequestered)	Covalently bound	Label incorporation into biomass
Formation from	Parent substance + transformation products	Parent substance + transformation products	Ultimate degradation and mineralisation
Evidencing	Identification of parent substance + transformation products	Identification of cleavage products, (highly difficult)	Label in biomarkers
Formation processes	van der Waals, hydrophobic interactions, ionic/ion–dipole/dipole–dipole forces, π–π interactions, ligand exchange, charge transfer, H-bonds	C–C, C–N, C–O–C, ester bonds (covalent bonds in general)	Microbial degradation
Stability	Low–high	High	Not relevant
Release probability of parent or transformation/degradation products	Low–high	Low	Not relevant
Extraction methods	‘Mild’–‘harsh’ for parent and transformation products	Cleavage methods for bonds of parent substances and transformation products	Biomarker: PLFA, amino acids, amino sugars
Analytical method	Typical methods used with radioisotope labelled derivatives include radio-HPLC–UV, radio-TLC, LSC, TopCount, radio-HPLC–MS, oxidative combustion followed by LSC; for additional, structural information, size exclusion chromatography or spectroscopic methods like NMR can be applied. Alternatively to radioisotopes, also stable isotope labeling can be applied with subsequent GC–MS or LC–MS analyses		GC–MS, HPLC–MS, 2D-thin layer chromatography
Cleavage methods	Silylation	Hydrolysis by KOH (ester-bonds), BBr_3_ (ether bonds), RuO_4_, (C–C bonds), TMAH (tetramethyl-ammonium hydroxide)	Not relevant
Assessment of remobilization potentials	For both types of NER Physical treatments: simulation of heavy rain events, hot water extraction, freeze/thaw cycling, grinding, wet/dry cycling Chemical treatments: pH changes, long-term Tenax extraction, changes in ionic strength, hydrolysis in the presence of Na^18^OH or H_2_^18^O Biological treatments: application of oxidative and other enzymes with release potential like peroxidases, laccases, and gluthathione-*S*-transferases, treatment with soil feeding organisms		Not relevant

### Methods to extract and identify NER, limitations of the test methods and technical challenges

The following sequence of extraction procedures is proposed to prepare an environmental matrix (soil, sediment, biological tissue) that, after removing the extractable fractions, only contains NER. If abiotically formed NER, i.e., in studies with sterilized matrix, are much lower in the control than biotically formed NER in a degradation test, this gives a clear indication on NER from degradation products or even from bioNER. If bioNER are actually formed then they can be counted as metabolized substance, in addition to CO_2_ and can, thus, be added to the degraded amount. If DT50 values are much lower than real DegT50 values with low amounts of resulting CO_2_, there is a strong indication for dissipation with formation of type I and II NER. In this case, the DegT50 value should be used in for the P/vP assessment [9].

Table 1 presents the properties of NER and the methodologies that can be used for the identification of the three NER types.

The following stepwise approach (Fig. 1) is proposed for the assessment of extractable residues and the characterisation of NERs in environmental matrices.

Step 1 shows the sequence of three extraction steps: aqueous salt/buffer extraction (bioavailable fraction), solvent/water mixtures (potentially bioaccessible, readily desorbable fraction), and finally exhaustive extraction using Soxhlet, ASE/PLE, SFE or MAE to release the extractable fractions (total extractable, remobilisable fractions). This sequence will leave only NER remaining in the matrix, which are defined here as total NER and can be subsequently used to assess and differentiate type I, II, and type III NER (Step 2). The extraction strategy should be substance tailored, e.g., by choosing appropriate solvents (see below).

#### Determination of extractable fractions (see Fig. 1, Step 1)

##### Use of aqueous solutions to determine the amount of residues being easily desorbable

Aqueous solutions will extract residues that are directly bioavailable for organisms living in the matrix, e.g., soil or sediment. A diluted CaCl_2_ solution, e.g., 0.01 M, is a suitable solvent for that purpose as the molarity and ionic strength resemble those of the soil pore water [56, 57]. An initial 0.01 M CaCl_2_ extraction should be performed at every sampling interval. CaCl_2_ extraction has been applied to correlate simazine residue bioavailability. Simazine sorption to soil increased with aging and amounts of simazine extracted by 0.01 M CaCl_2_ were clearly correlated with amounts of simazine mineralized by a simazine-mineralizing bacterium [58]. Similarly, CaCl_2_ extraction mimicked the bioavailable fractions of indaziflam, carbendazim and sulfadiazine in soil [59–61]. Aqueous solutions of nitrate or acetate salts can also been used for extraction [62–64].

##### Use of organic solvent mixtures to extract thoroughly the matrix

Available residues should be sequentially extracted at ambient temperature with carefully selected aqueous:organic solvent mixtures (e.g., 50:50 or 20:80 water:acetonitrile; v:v), which at times may be modified with minute amounts (0.1–2.5% v/v) of formic acid, acetic acid and/or ammonia in order to enhance the solubility of the xenobiotic and/or its transformation products. Elevated temperatures are avoided for the initial extracts of the samples. Samples will be extracted for prolonged time periods (4–24 h) using physical agitation, e.g., shaking. Ultra-sonication may enhance the extraction efficiency, but the temperature of the sample should then be monitored. In studies with radiolabeled compounds, sequential extractions should be performed until < 5% of the radioactivity released from the first extraction is obtained. This usually occurs between three and five extractions with one solvent system [65].

The selection of the proper organic solvents is a critical step. The physico-chemical properties of the analyte, i.e., its volatility, water solubility, the solubility in the organic solvent to be used, the pKa, and the stability, as well as the test matrix properties (such as the moisture and organic matter content of soils and sediments), must be considered [66]. Properties of some extraction solvents and their relations to properties of analytes are given in Additional file 1: Tables S1 and S2. Some examples are presented in Table 2 how to remove extractable fractions from environmental matrices to obtain non-extractable residues.

**Table 2:** Some examples of conditions to remove extractable fractions from environmental matrices to obtain non-extractable residues

Analyte	Matrix	Solvents and methods to remove extractable fractions	References
Cyprodinil	Soil	Methanol	[35, 45, 71–73]
Simazine	Compost	Methanol:water 9:1 (v:v)	[33]
Sulfadiazine	Soil	Ethanol:water 9:1 (v:v), Soxhlet	[46, 61]
MCPA	Soil, clay	Methanol; dichloromethane, Soxhlet	[74]
Nonylphenol	Soil, clay	Methanol; dichloromethane, Soxhlet	[36]
Nonylphenol	Soil	Methanol and ethylacetate	[75]
Difloxacin	Soil	Ethylacetate:methanol:water:ammonia 63:25:9:3 (v:v:v:v), ASE	[44]
Metalaxyl	Soil, sand, silt, clay	Methanol, Soxhlet	[76]
Clodinafop-propargyl	Sediment	Acetonitrile, Soxhlet	[77]
3,4-Dichloroaniline	Sediment	Methanol	[78]
Isoproturon	Soil	Methanol, ASE	[79]
Tetrabromobisphenol A	Soil	Methanol; methanol and ethylacetate	[80–82]
Phenanthrene	Soil	Dichloromethane and acetone	[83]
Cypermethrin	Soil	Acetonitrile + water 7 + 3 (v + v)	[84]
Cypermethrin	Soil	Cyclohexane:acetone, 1:1 (v:v)	[8]
Ciprofloxacin	Soil	Ethylacetate, methanol, ammoniumhydroxide, ASE	[85]
Imidacloprid	Soil	Methanol, water, HCl, Soxhlet	[86]
Imidacloprid	Soil	Acetonitrile, water, MAE	[87]
Carbendazim	Soil	Methanol, Soxhlet	[88]
Isoproturon	Soil	Acetone, Soxhlet	[89]
Metalaxyl	Soil	Acetonitrile, water, ASE	[90]

Pure organic solvents should be avoided in the first extraction steps because molecules distributed in the interlayers of clay particles in soil may be entrapped by shrinking of the clay when water is removed. Therefore, in the first extraction steps, water-miscible organic solvents should be mixed with small volumes of water, followed by exhaustive extraction pure solvents (or solvent mixtures). Extracts should be combined and concentrated prior to radio-profiling for instance by radio-HPLC or radio-TLC.

Since transformation products of chemicals usually differ from the parent compound in terms of polarity (most often more polar, sometimes less polar) and chemical reactivity (as well as ecotoxicity), extraction procedures have to be developed during the course of a degradation study. An effective extraction solvent for the parent compound is usually not effective for transformation/degradation products. It is, therefore, not possible to define optimal extraction conditions at the beginning of the study and to keep this procedure for aged samples; exemptions are of course possible. Both polar and nonpolar solvents, or miscible solvent mixtures should be tested for extraction depending on the nature of the residues.

While any changes of the parent substance and of major transformation/degradation products by the chosen extraction method can be tested by corresponding control experiments, its effect regarding structural changes of the matrix is much more difficult to assess. Soil scientists claim that soil structure is even changed by different moisture contents and the quality of percolating water [67, 68]. Therefore, any method to extract soil, even under “mild” conditions, will lead to some structural changes. This holds especially for using organic solvent mixtures, both at room temperature and elevated temperatures.

##### Exhaustive extraction

Soxhlet extraction, accelerated solvent extraction (ASE) or pressurized liquid extraction (PLE), supercritical fluid extraction (SFE), or microwave-assisted extraction (MAE) of the particulate matrix remaining should subsequently be applied, eventually using suitable modifier solvents, to release part of the molecules strongly adsorbed to the matrix [69]. If feasible, extracts can be analyzed by the same methods used for the previous extractions.

Strongly acidic and alkaline solvents for release of the extractable fraction (with simultaneous partial humic compound extraction) need to be avoided since severe structural changes regarding the inorganic (acids) and organic (alkali) components of the matrix will occur.

The approach of the described extraction steps “Use of aqueous solutions to determine the amount of residues being easily desorbable”, “Use of organic solvent mixtures to extract thoroughly the matrix” and “Exhaustive extraction” follows in principle that of Ortega-Calvo et al. [70] who suggests to use the extraction sequence aqueous solutions, passive sampling extractions (e.g., TENAX), organic solvents at room temperature and at elevated temperature (e.g., ASE), to obtain the matrix containing only NER. Each of the fractions obtained by the above extraction steps can be used to analyze the amounts and to identify the structures of the extractable fractions. The residues remaining after the extractions in the matrix are defined as non-extractable residues (NER), which may include also bioNER (type III). These NER, thus, should be analyzed for the different types of NER.

#### Differentiation of NER types (see Fig. 1, Step 2)

For performing Step 2 extractions, samples must be splitted into two aliquots or sub-samples because silylation [step “Differentiation and quantification of type I and type II NER by silylation of the matrix”) and amino acid extraction [step “Quantification of type III NER (bioNER)”] are considered to be “destructive” methods and cannot be applied to the same sample sequentially.

##### Differentiation and quantification of type I and type II NER by silylation of the matrix

Silylation is a gentle derivatization method and has been used for decades in synthetic and analytical chemistry. In the environmental context, this pragmatic approach can be applied for quantification of the two NER types I and II. However, it does not provide information about the chemical identity of the NER as long as the released residues are not characterized, for instance, by mass spectroscopic approaches. Each fraction derived from these procedures may also contain bioNER. It cannot be excluded that by silylation, some residual type I NER will remain in the matrix, although this seems rather unlikely, but should be investigated by repeating the silylation step. If some residual type I NER would remain after this derivatization method, this would lead to an underestimation of type I NER, which are basically the releasable part, and an overestimation of type II NER.

Silylation will replace the exchangeable hydrogen atoms of functional groups in the organic matrix—e.g., carboxylic, hydroxy or amino groups—with trimethylsilyl groups [91]. The silylation breaks hydrogen bonds between polar functional groups and changes the hydrophilicity of organic matter, resulting in a partial disintegration of the humic substances into smaller fragments, which have been held together in supramolecular aggregates by noncovalent interactions in the original sample. Labile test substances may also be destroyed by this method but this can be checked for the respective compounds. If NER are entrapped in the matrix (type I NER candidates), they are released after silylation, while NER formed by covalent binding (type II NER candidates) remain bound to the matrix. Both fractions can be quantified when radioactively labelled chemicals have been used. However, for the final determination of the type I or II NER extent, the amounts of included bioNER need to be evaluated. In the type I NER containing fraction, this can either be done by quantification of the parent substances and transformation products, e.g., by MS, or the amount of bioNER [53]. The type II NER containing fraction needs to be calculated by the total NER after exhaustive extraction minus total bioNER and identified type I NER. These data can be used as endpoint for quantification of type I and type II NER. The experimental steps in the silylation procedure were described in detail, e.g., by [33] or in a slightly modified form [83].

##### Quantification of type III NER (bioNER)

Both labeling with radioactive (^14^C) [53] and stable isotopes (^13^C, ^15^N) [12] has been successfully applied to quantify the amounts of type III NER using the extraction method. Basically, the environmental matrix, e.g., soil, is hydrolyzed by concentrated HCl at elevated temperature. The matrix and particularly the proteins are destroyed under such harsh conditions and the hydrolyzed extract contains the released amino acids [38, 40–42, 85]. Based on amino acid detection of hydrolyzed proteins, the total living biomass (which is bioNER) of short-term experiments should be calculated by multiplying the amino acid amount by the factor of 2, since the amount of proteins in living microbial biomass is generally around 50% [92]. During microbial turnover of microbial biomass, however, the ratio bioNER to proteins decreases and approaches 1.11 for long-term experiments (≥ 120 days) [54].

##### Uncertainties and limitations of the methods

Methodological uncertainties come primarily from the procedures for removing the extractable residues to obtain the matrix containing only NER. Taking the paper from Barriuso et al. [11], the amounts of NER of pesticides vary strongly depending on the extraction procedures, with the largest variations of a factor up to 10. To give few examples: the amounts of NER of imazosulfuron vary between 19 and 68% of the applied radioactivity, that of propoxycarbazone between 6 and 65%, of propiconazole between 4 and 48%. Another methodological uncertainty is the extraction procedures for investigation of NER (silylation for type I and type II NER differentiation and acidic hydrolysis for bioNER quantification): it is likely that silylation extracts and residues besides xenobiotic residues also contain bioNER, especially for compounds that are readily biodegraded. Therefore, type I non-extractable residues need to be investigated to address this uncertainty, but a method for analytical differentiation needs to be developed. Chemical analysis of type II NER, which are strongly bound and not releasable under physiological, natural conditions, is, however, not possible, i.e., the uncertainty of the apportionment of xenobiotic and biogenic residues in these fraction cannot be settled.

As a further uncertainty, neither the silylation method to distinguish type I and type II NER nor the method to identify bioNER type III have been standardized but rather represent methods derived from basic research. Structural identification of type I and type II residues is a technical challenge and laborious. As a pragmatic approach, the released amount of NER after silylation can be taken as type I NER, that remaining in the matrix as type II NER. Assuming that the relative amount of type III NER, which is determined independently by the described acidic hydrolysis method, is the same also in the type I and type II NER fractions, it is possible to estimate the absolute amounts of types I and II.

#### Options for the assessment of the potential remobilization of total NER

Many studies reveal that NER may become released under natural environmental conditions, such as the microbial activity in the rhizosphere of plants or in the digestive tract of animals, but the released residues enter a matrix with degrading activity and may subsequently be transformed or partly mineralized. Release of NER has been observed also by applying artificial conditions that, however, will never (e.g., EDTA addition) or only rarely happen under natural conditions.

Several authors investigated the stability of NER formed during microbial turnover of environmental contaminants such as PAH [93] and TNT [94, 95]. NER derived from ^14^C-labelled anthracene or the explosive ^14^C-2,4,6-trinitrotoluene (TNT) in soil were analyzed after simulation of extreme physical, chemical or biological conditions in a systematic manner. These authors used the following treatments for the residue containing soils.

i. Physical treatment for simulating climatic effects by freezing and thawing [96, 97], wetting and drying or by changing the soil texture via ploughing or grinding [93];
ii. Chemical treatment, the extraction of soil with the metal complexing agent EDTA [98] for estimating the effects of bivalent cations on the aggregation of macromolecular soil organic compounds and the extraction of soil with hot or acidified H_2_O simulating a millennial rain event and the acid rain impact on the release of NER [99].
iii. Biological treatment, the simulation of increased turnover of SOM and the NER by addition of compost [23, 100] or incubation of the soil containing NER with ligninolytic fungi [101–103].

It has to be taken in mind that remobilization experiments are an operational approach, and the absence of remobilization is no conclusive evidence for covalent binding.

### The microbial turnover to biomass (MTB) for predicting NER formation

Chemicals that are easily biodegradable show high mineralization rates and are, thus, prone to formation of biogenic NER, whereas those which are poorly biodegradable (persistent) with low mineralization rates will mainly form type I and type II NER [12].

A clear correlation has been recently established between released CO_2_ (as indicator of microbial activity and mineralisation), biomass yield, and biogenic NER formation [104]. This relation can be used as a screening tool or indicator for bioNER formation in a two-step process: first, the theoretical growth yield is estimated from thermodynamics and molecule structure. Second, the yield together with the information about CO_2_ production (determined experimentally in a biodegradation test) is used to calculate the microbial biomass growth. If the experiment is long term, then also the biomass turnover in the microbial food chain is considered. The sum of living and dead biomass plus organic matter originating from this dead biomass contributes to bioNER [54].

Microbial growth and degradation of the test chemicals lead to the incorporation of labeled carbon into the microbial mass, resulting in biogenic NER. The MTB approach needs minimum input data (Gibbs free energies of products and educts, molar mass, the empirical formula of the chemical, and the number of CH bonds) which are readily available. The microbial growth yields of 40 organic chemicals of environmental concern (including 31 pesticides) were recently estimated. The results were compared to experimental values and the results of other methods for yield estimation that are available in the literature. With the theoretical biomass yield and using the released CO_2_ as a measure for microbial activity, a range for the formation of biogenic NER could be predicted. For the majority of the pesticides, a considerable fraction of the NER was estimated to be biogenic.

The MTB yield estimate has shown the best performance for the yield prediction of xenobiotics but still had a mean average error (in comparison to experimental data which may also have some error) of 49% with both overestimation and underestimation [105]; the high deviation is due to failure in few cases, and reasons for the failure could be identified. Validation with ^13^C-studies showed good agreement to measured growth yields of 2,4-D and ibuprofen [54] which form much higher bioNER than type I and II NER; more such data will be helpful and are currently under production.

The MTB tool can be used in the persistence assessment as a screening tool for the estimation of the likeliness of type III NER versus type I and II (xenoNER). The MTB yield method is quite new and experience with tested chemicals is still limited. Until sufficient (and supporting) experience has been gained, the method should only be used as an indicator and not as a definitive proof for bioNER formation; if the MTB method indicates relevant bioNER formation, then the formation of bioNER should be tested experimentally.

**Fig. 2:**
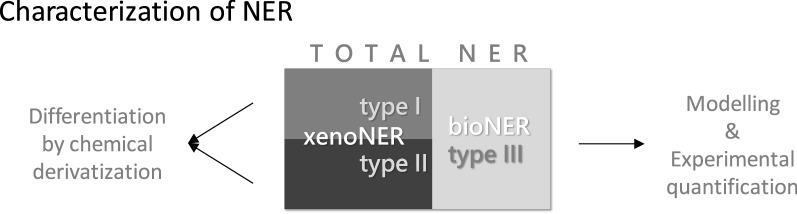
NER characterization concept. Experimental and modeling steps to differentiate the three types of NER

A conceptual sketch on the characterization of NER is provided in Fig. 2.

## Concepts for assessing the role of NER in the regulatory context

### NER in P assessment

As shown, the experimental and theoretical differentiation of NER types is nowadays possible and should be considered in the persistence assessment of chemicals. If the likeliness for the formation of biogenic NER is high (presumably derived from the in silico assessment with MTB and the confirming mineralisation in the fate assessment), the consideration of NER as parent substances or primary transformation/degradation products is not justified and here the analysis of bioNER is suggested. For the remaining NER of type I (for type II only if there are indications for remobilisation potential), a remobilization assessment is suggested (see Table 1). A guide value for remobilization of NER can be given: only if no or very low amounts of residues are released (for soil organic matter, < 2% C/a), we consider this as no remobilization. The turnover of 2% C/year is the average respiration of a living soil including soil organic matter under aerobic condition [106, 107]. Conant et al. [106]. assumed that 5–15% of SOM, i.e., the readily degradable, “fresh” organic carbon pool, is decomposed within months to years, 40–50% of SOM, the intermediate pool, within years to decades, and 40–50% of SOM, the stable “old” organic matter, within decades to centuries.

Based on the present scientific analysis of the NER formation and the screening of the available related documents, it can be stated that characterization of NER can be embedded in the general persistence assessment of all chemicals. We propose to generally consider unknown total NER as remobilizable parent or transformation products, if no additional information is available. If clear indications for bioNER and also for covalently bound type II NER are available (unless there exist indications for a remobilization potential), these NER can be considered as ‘safe sink’. Covalently bound NER, in particular if multi-covalently bound, are believed to have very low remobilization potential since such bonds are considered to be rather stable under physiological conditions [91, 93, 108].

The MTB approach [54] is suggested to be used for the general estimation of the biomass yield and the bioNER formation potential to obtain information before setting required OECD degradation or fate simulation tests. Indications for bioNER can be cross-validated by assessing CO_2_ formation in degradation experiments, or the bioNER can be analytically confirmed. Vice versa, dominantly NER type I or II forming chemicals can be identified, which may not need further evaluation of bioNER. This approach is very important for not wasting time with inappropriate testing of a certain chemical without a specific NER focus and is also helpful for the interpretation of results derived from these tests. The general concept of the MTB application is to consider the total NER minus potential bioNER as the amount of type I + II NER. Type I is considered to include remobilization potential; whereas type II is considered to be irreversibly bound (unless there are indications for the opposite). The MTB yield estimation can also be applied to gain information about P indication—very low biomass yields are an indicator for persistence.

### How to deal with complex mixtures and natural compounds?

Persistence assessment of mixtures and substances of unknown or variable composition, complex reaction products or biological materials (UVCBs) is a challenge (ECHA_2017_R.11 Section R11.4.2.2). Approaches rely on constituent profiling, identification of known constituents, or block profiling (functional blocks), if no known constituent can be identified. Our proposed approach for NER characterisation can only be applied for ‘identified constituents’ generally based on the application of isotope labelled compounds. In a broader sense, it may also be applied to mixtures and substances of unknown composition as long as known compounds or constituent blocks, or the compounds of most critical concern can be identified. If the most critical constituent can be identified, the general suggested approach of NER characterisation is valid. If feasible, the fate and turnover of the compounds in the mixture should always be compared to the behavior of the pure compounds to evaluate additional effects caused by the mixture. If only the ‘whole substance approach’ is applicable without supply of any labelled compound, the NER cannot be quantified.

The MTB approach can be applied to each constituent separately, or to a block of constituents, if a common chemical structure can be identified. Then a lumped biomass yield can be calculated. If the physical and chemical properties of the mixtures are highly different from the known constituents, for example for specific compounds in non-aqueous phase liquids (NAPLs), the fate and distribution behavior can be affected [12, 109]. NAPLs may cause mass transfer problems and result in not fully developed biomass yields.

## Recommendations for further research

There are multiple causes of diverging results in fate studies of chemicals even for one compound in one test system and this is actually a very difficile problem and cannot be solved with general considerations. Adding an additional analytical parameter like NER makes the situation even more complex. Therefore, only a weight of evidence evaluation of each test result is possible and needs to be taken with care and expertise.

### Types I and II NER (xenoNER)

The following investigations should be performed with environmental matrices which have been thoroughly extracted and contain NER only. The release of parent substances and transformation/degradation products should be systematically studied (“Options for the assessment of the potential remobilisation of total NER” section), also with respect to the question whether this release is associated with the degradation rate of natural soil organic matter.

In cases where type II NER has been unambiguously demonstrated, cleaving of covalent ester-bonds by labelled water (H_2_^18^O) or sodium hydroxide (Na^18^OH) can clearly prove covalent binding as shown for the transformation/degradation products of DDT and metalaxyl which were bound by ester-linkage to humic matter [52]. However, other types of covalent bonds, like Michael adducts or Schiff base adducts, cannot be investigated by this methodology.

### Type III NER (biogenic residues)

Predictions of bioNER by MTB modeling need to be validated by experimental investigations of more chemical substances. Also, the effect of repeated applications of a chemical to soil (as in case of spray series of pesticides) on the formation of type III NER should be further investigated: adaptation of soil microorganisms and accelerated degradation has been described [110–112] but so far not with respect to NER formation. Principally, such effects can be considered by the MTB method.

### General issues

There is still a set of issues that remain to be investigated regarding NER characterization.

- There is urgent need for standardization of the NER extraction methods. Regular ringtests should be performed to validate the proposed silylation technique for distinction of type I and type II NER as well as the hydrolysis method for determining bioNER. The efficiency of the silylation method to release all type I NER should be tested, e.g., by repeating silylation of the matrix.
- There is also need for standardization of NER remobilization assessment methods (“Options for the assessment of the potential remobilisation of total NER” section), which are also less sufficiently described in the literature. Experiments should be performed with a set of chemicals covering various functional groups.
- The relationship between extraction technique and bioavailability: there are currently several methods available to assess the bioavailability or bioaccessibility of chemicals in environmental matrices [66]; however, the release and the accessibility on the long run or under changes of the environmental conditions are still an open question.
- The potential correlation of the bioavailability (e.g., by passive sampling) and ecotoxicity of NER, especially of those chemicals forming significant amounts of type I NER should be studied.
- A special case of NER may be the conjugates of chemicals (pharmaceuticals, pesticides) in NER assessment, in particular if entering the environment by activated sludge or manure. These are no NER in sensu stricto but they may also become associated with particulate matter in environmental systems. The remobilization potential of such compounds needs to be tested.
- Special consideration has to be given to the class of poorly water-soluble substances with log Kow values above about 6, which have a high tendency to adsorb to particulate matter like soil or sediment. These compounds quickly partition from the aqueous phase to pores of the humic matter becoming strongly sorbed or sequestered (type I NER). Even if the inherent biodegradability is high, they become rather persistent in the sequestered state if desorption rates are very slow. Persistence is clearly influenced by partitioning to particulate matrices as has been shown by many examples: even biodegradable substances like proteins persist after immobilization to a solid matrix.
- Concepts to describe the competing kinetics of adsorption, sequestration and biodegradation kinetics are available, and at least one model exists that can, with reasonable input data, estimate simultaneous formation of type I NER and type III bioNER, namely the unified model for sorption and biodegradation [12, 54]. However, few studies have been performed where detailed experimental data has been used for comparison to simulation results, and more research is helpful for confirmation and verification of model concept and output. Similarly, mathematical tools that simulate the outcome of OECD tests, e.g., [113–115], would be useful both for interpretation and confirmation of the test results. The development and test of such models are recommended.

## Additional file

**Additional file 1: Table S1.** Properties of typical organic solvents and water. **Table S2.** Relative polarity of chemical classes and examples of typical extraction solvents; also mixtures of solvents can be used. The selection is not exclusive and several solvents listed cover a range of chemical classes to be extracted.

## Abbreviations

ASE: accelerated solvent extraction (extraction with the option of elevated temperature and pressure (cf. PLE)
BBr_3_: borontribromide
bioNER: biogenic NER
DT50: time taken for 50% of substance to disappear from a compartment by dissipation processes (dissipation half-life)
DegT50: time taken for 50% of substance to disappear from a compartment due to degradation processes alone (degradation half-life)
ECHA: European Chemicals Agency
GC-MS: gas chromatography coupled to mass spectrometry detection
HPLC–MS, HPLC–UV: high performance liquid chromatography coupled to mass spectrometry detection or UV detector, respectively
KOH: potassium hydroxide
LSC: a method to quantify radioactivity by mixing a radioactive sample with a liquid scintillator in a solvent and counting the resulting photon emissions. The photons are released from scintillator molecules after excitation by the radioactive material (such as beta particles emission from ^14^C-labeled material). Alternatively, a radioactive sample in a solvent can be passed through a matrix with immobilized scintillators
MAE: microwave-assisted extraction
MTB: microbial turnover to biomass (model to predict formation of bioNER)
NER: non-extractable residues
NER type I: sequestered, entrapped NER
NER type II: covalently bound NER
NER type III: biogenic NER (bioNER)
NMR: nuclear magnetic resonance
PLE: pressurized liquid extraction at elevated pressure and temperature. This method of extraction is equivalent to the accelerated solvent extraction (ASE)
PLFA: phospholipid fatty acids, components of every cell membrane
Radio-HPLC: high-performance liquid chromatography coupled to a radioactivity detector, for instance for profiling ^14^C-labeled residues in degradation studies
Radio-TLC: thin layer chromatography and subsequent analysis of radiolabeled residues, e.g., ^14^C-residues, on the plate by a radioactivity detector
RuO_4_: ruthenium tetroxide
SFE: supercritical fluid extraction; extraction of mostly a solid matrix, like soil or sediment, using supercritical fluids as extractants. Supercritical fluids share the properties of liquids (with high dissolving power) and gases (with high diffusion coefficients) above a certain temperature and pressure. Often, carbon dioxide is used as supercritical fluid, sometimes modified by co-solvents like ethanol, because the critical temperature of CO_2_ (31 °C) and critical pressure (74 bar) are easily achieved
SOM: soil organic matter
xenoNER: NER containing xenobiotic molecules, i.e., the parent substance and transformation products
TLC: thin layer chromatography, for instance two-dimensional (2D-TLC)
TMAH: tetramethylammonium hydroxide, derivatization agents
UVCB: substances of unknown or variable composition, complex reaction products or biological materials
