# Supplementary material for: A unified approach for including non-extractable residues (NER) of chemicals and pesticides in the assessment of persistence
Source: Environ Sci Eur. 2018 Dec 17;30(1):51. doi: 10.1186/s12302-018-0181-x (PMC6297198; doi:10.1186/s12302-018-0181-x)
Supplement: Supplementary file 1 — Additional file 1: Table S1. Properties of typical organic solvents and water. Table S2. Relative polarity of chemical classes and examples of typical extraction solvents; also mixtures of solvents can be used. The selection is not exclusive and several solvents listed cover a range of chemical classes to be extracted. [file 12302_2018_181_MOESM1_ESM.docx]

A Unified Approach for including Non-Extractable Residues (NER) of Chemicals and Pesticides in the Assessment of Persistence

Andreas Schäffer^1^, Matthias Kästner^2^, Stefan Trapp^3^

^1^ RWTH Aachen University, Institute for Environmental Research, Worringerweg 1, 52074 Aachen, Germany

^2^ UFZ, Helmholtz Centre for Environmental Research, Department Environmental Biotechnology, Permoserstraße15, 04318 Leipzig, Germany

^3^ Technical University of Denmark, Department of Environmental Engineering, Bygningstorvet bd. 115, 2800 Kongens Lyngby, Denmark

*Correspondence to: [andreas.schaeffer@bio5.rwth-aachen.de](mailto:andreas.schaeffer@bio5.rwth-aachen.de)

Additional file

Table S1: Properties of typical organic solvents and water.

| **Solvent** | **Boiling point (° C)** | **Snyder polarity index*** |
| --- | --- | --- |
| Pentane | 36 | 0.0 |
| Heptane | 98 | 0.1 |
| Hexane | 69 | 0.1 |
| Cyclohexane | 81 | 0.2 |
| Toluene | 111 | 2.4 |
| o-Dichlorobenzene | 180 | 2.7 |
| Ethyl Ether | 35 | 2.8 |
| Dichloromethane | 40 | 3.1 |
| n-Butyl Alcohol | 118 | 3.9 |
| Isopropyl Alcohol | 82 | 3.9 |
| Tetrahydrofuran | 66 | 4 |
| Chloroform | 61 | 4.1 |
| Ethyl Acetate | 77 | 4.4 |
| 1,4-Dioxane | 101 | 4.8 |
| Acetone | 56 | 5.1 |
| Methanol | 65 | 5.1 |
| Ethyl Alcohol | 78 | 5.2 |
| Acetonitrile | 82 | 5.8 |
| Dimethylformamide | 153 | 6.4 |
| Water | 100 | 10.2 |

* Snyder polarity index: Solvent polarity is a function of the dipole moment, proton acceptor or donor properties of chemicals and further properties. The Snyder's polarity index ranks solvents according to a summation of these properties. The higher the polarity index, the more polar the solvent. Snyder's paper was published in Journal of Chromatography A 92, 223-230 (1974).

Table S2: Relative polarity of chemical classes and examples of typical extraction solvents; also mixtures of solvents can be used. The selection is not exclusive and several solvents listed cover a range of chemical classes to be extracted.

| **Relative polarity ↓** | **Chemical class** | **Compounds** | **Typical solvents** |
| --- | --- | --- | --- |
| NONPOLAR | R-H | Alkanes | Hexane |
|  | Ar-H | Aromatics | Toluene, benzene |
|  | R-O-R | Ethers | Diethyl ether |
|  | R-Hal | Alkyl halides | Tetrachloromethane |
|  | R-COOR | Esters | Ethyl acetate |
|  | R-CO-R | Aldehydes, Ketones | Acetone, acetonitrile |
|  | R-NH_2_ | Amines | Acetonitrile, triethylamine* |
|  | R-OH | Alcohols | Methanol, ethanol, acetonitrile |
|  | R-CONHR | Amides | Acetonitrile, dimethylformamide* |
|  | R-COOH | Carboxylic acids | Acetonitrile-water mixtures, diluted acetic acid |
| POLAR | H-OH | Water | Water |

* Triethylamine is an ion-pairing reagent and strong base and strips functional groups from the inside of reverse-phase HPLC columns and the surface of silica TLC plates. Dimethylformamide is a viscous solvent with a high boiling point that may also damage the solid phase of chromatographic columns in subsequent analyses. Therefore, both solvents should be used only as modifying additives.
